# Colour triad in rare-earth doped glass: a step toward volumetric displays

**DOI:** 10.1038/s41377-025-01763-8

**Published:** 2025-02-13

**Authors:** Yiyuan Zhu, Renren Deng

**Affiliations:** 1 Department of Medical Oncology, The First Affiliated Hospital, School of Medicine, Zhejiang University, Hangzhou, 310003 China; 2 State Key Laboratory of Silicon and Advanced Semiconductor Materials, Institute for Composites Science Innovation, School of Materials Science and Engineering, Zhejiang University, Hangzhou, 310058 China

**Keywords:** Imaging and sensing, Displays

## Abstract

Full-colour tuning in rare-earth doped monolithic tellurite glass is realised via excitation pulse modulation, enabling a novel platform for laser-based transparent displays. This advancement demonstrates the potential of upconversion emission for future display technologies.

## Abstract (web summary)

Full-colour tuning in rare-earth doped monolithic tellurite glass is realised via excitation pulse modulation, enabling a novel platform for laser-based transparent displays.

## Graphical abstract

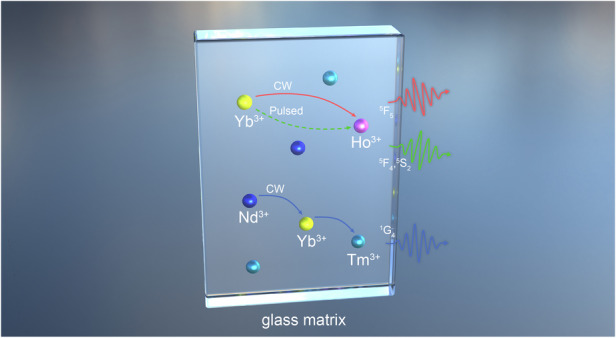

The quest for more vibrant and immersive display technologies has catalysed remarkable progress over the decades. From cathode-ray tubes (CRTs) to micro light-emitting diodes (microLEDs), each generation of display technology has sought to deliver richer colours and sharper images. As flat panels approach their zenith, the focus is shifting toward volumetric three-dimensional (3D) displays, promising a leap into more dynamic and interactive visualisations^1–3^. However, the path to practical volumetric displays is paved with challenges in light manipulation, material design, and fabrication. Research in this field remains in its infancy, highlighting a crucial area for future exploration and innovation.

Volumetric 3D displays create images within a physical space, allowing viewers to observe content from multiple angles without the need for special eyewear. Extensive research has explored various techniques, including swept-volume displays, holographic displays, and static volumetric displays^3–9^. Among the techniques explored, upconversion-based volumetric displays, leveraging rare earth (RE) doped phosphors, stand out as a highly promising technique. This technique utilizes invisible near-infrared excitation to produce visible-spectrum emissions, enabling full-colour 3D imaging while preventing the graphic formation from excitation interference within volume pixels^6,10–13^. However, achieving scalable 3D colour displays remains a challenge. Key issues include inefficient upconversion luminescence, colour purity degradation due to cross-relaxation effects, and the difficulty of precise emission colour control within a 3D space.

Now, writing in *Light Science & Application*, Ekim et al. take an important step forward by integrating laser-modulated emission colour tuning into a scalable monolithic tellurite glass matrix. Their work bridges fundamental principles of upconversion luminescence with practical material design, offering a pathway to future volumetric display applications. The tellurite glass system is co-doped with Ho^3+^, Tm^3+^, Yb^3+^, and Nd^3+^ ions, each contributing to the RGB colour spectrum via upconversion mechanisms^14^.

Specifically, a continuous-wave (CW) 980 nm laser excites Yb^3+^ ions, which transfer energy to Ho^3+^ ions, activating the ^5^F_5_ state of Ho^3+^ for red emission at 661 nm. Pulsed 980 nm excitation predominantly excites the ^5^F_4_ and ^5^S_2_ states of Ho^3+^ ions, leading to green emission at 548 nm. A CW 808 nm laser excites Nd^3+^ ions, which transfer energy through Yb^3+^ to Tm^3+^ ions in the ^1^G_4_ state, producing blue colour emission at 480 nm. This laser-tuned approach enables precise, dynamic control of RGB emissions by modulating excitation parameters, such as excitation wavelength and pulse width. As a result, the system achieves the creation of moving, rotating, and expanding 3D images within the glass volume, representing a significant milestone for volumetric display technologies (Fig. 1).

**Fig. 1:**
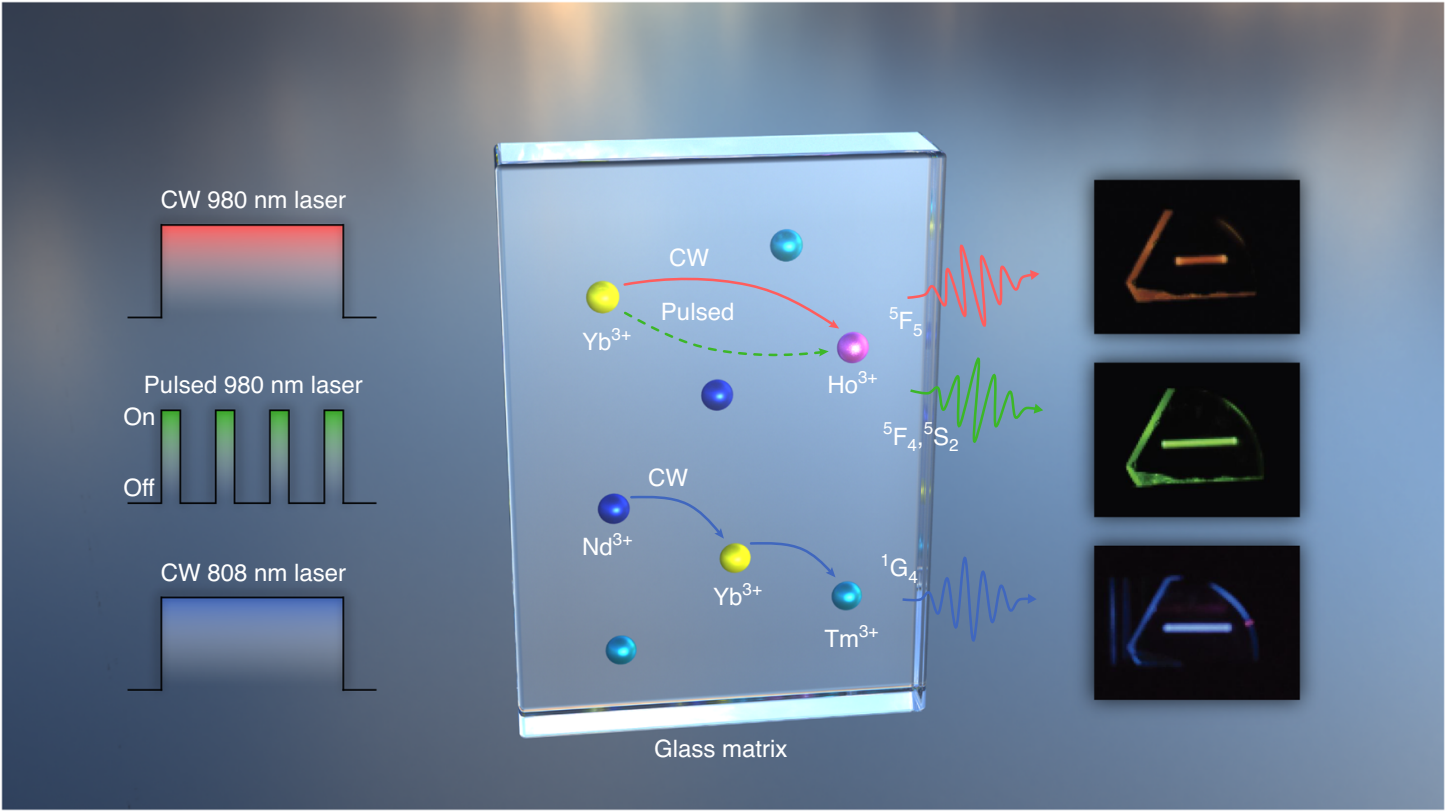
Schematic representation of laser excitation-modulated RGB colours in rare-earth doped monolithic tellurite glass

The monolithic structure of the tellurite glass matrix avoids the complexity of fabricating core-shell nanocrystals, offering scalability for industrial production. However, achieving optimal colour purity requires careful selection and precise proportioning of rare-earth ions to mitigate luminescence quenching and unwanted cross-relaxation. Enhancing the efficiency of blue emission, which remains a challenge, and minimizing non-primary colour emissions are critical areas for improvement. Developing additional strategies to refine excitation conditions and emission control will be essential for achieving the high colour purity necessary for advanced volumetric display applications.

Volumetric displays based on RE upconversion open up a wide range of potential applications. For example, transparent glass screens could display 3D molecular models in classrooms or integrate into architectural designs as interactive information panels. While these ideas remain conceptual, Ekim et al.’s work highlights the feasibility of such applications, underscoring the potential of upconversion-based volumetric displays. Recently, RE-based upconversion has transformed fields ranging from bioimaging to upconversion lasing^15^, and its application to volumetric displays marks an exciting frontier. By integrating advanced material design with precise excitation strategies, researchers are bringing the concept of dynamic 3D displays closer to reality. Continued exploration of materials and optimization methods will undoubtedly accelerate the journey toward commercial viability.
